# Biomass Porous Carbons Derived from Banana Peel Waste as Sustainable Anodes for Lithium-Ion Batteries

**DOI:** 10.3390/ma14205995

**Published:** 2021-10-12

**Authors:** Fernando Luna-Lama, Julián Morales, Alvaro Caballero

**Affiliations:** Departamento Química Inorgánica e Ingeniería Química, Instituto Universitario de Química Fina y Nanoquímica (IUNAN), Facultad de Ciencias, Universidad de Córdoba, 14071 Córdoba, Spain; q12lulaf@uco.es (F.L.-L.); iq1mopaj@uco.es (J.M.)

**Keywords:** banana peel waste, biomass, anode, Li-ion batteries

## Abstract

Disordered carbons derived from banana peel waste (BPW) were successfully obtained by employing a simple one-step activation/carbonization method. Different instrumental techniques were used to characterize the structural, morphological, and textural properties of the materials, including X-ray diffraction, thermogravimetric analysis, porosimetry and scanning electron microscopy with energy-dispersive X-ray spectroscopy. The chemical activation with different porogens (zinc chloride, potassium hydroxide and phosphoric acid) could be used to develop functional carbonaceous structures with high specific surface areas and significant quantities of pores. The BPW@H_3_PO_4_ carbon exhibited a high specific surface area (815 m^2^ g^−1^), chemical stability and good conductivity for use as an anode in lithium-ion batteries. After 200 cycles, this carbon delivered a reversible capacity of 272 mAh g^−1^ at 0.2 C, showing a notable retention capacity and good cycling performance even at high current densities, demonstrating its effectiveness and sustainability as an anode material for high-energy applications in Li-ion batteries.

## 1. Introduction

Given the growing concern regarding global warming and environmental issues derived from the greenhouse gas emissions from fossil fuel sources, new energy sources that are cleaner and more sustainable are emerging [1]. Within this context, energy storage systems based on lithium-ion batteries (LIBs) have attracted significant attention in industry in recent decades for use in portable devices, such as laptops, smartphones and other electronics, due to their good energy/power density, optimal cycle life and environmental friendliness [2]. So far, graphite has been the reference anode material in commercial LIBs because of its high electrochemical stability, low cost and availability [3,4]. Nevertheless, its limited lithium storage capacity (372 mAh g^−1^), poor rate performance and difficulty in recycling, while only at the research level [5], are not sufficient to meet the high energy demands of applications for the next generation of hybrid and electric vehicles [6].

Through the efforts of the scientific community to research and develop new anode materials, soft and hard carbons have appeared as promising replacements for graphite. Although soft or graphitizable carbons, such as porous carbons [7], carbon nanofibers [8], carbon nanotubes [9], and graphene [10], are widely used in LIBs because of their large specific surface areas, high conductivities and storage capacities, these materials involve complex and expensive synthetic processes that reduce their commercial applications [11]. In these syntheses, high cost and high purity raw materials (frequently petrochemical derivatives) are essential, in combination with non-simple techniques, such as arc discharge, laser ablation or chemical vapor deposition (CVD). In contrast, hard or non-graphitizable carbons, especially those based on biomasses, are receiving significant attention because of their controllable morphology and porosity, simple synthesis, vast availability and environmental friendliness [12,13,14]. There are a large number of biomass resources employed in LIBs, including bamboo [15], tea leaves [16], coffee grounds [17], rice straw [18], corn stalk [19], cherry pits [20], olive stones [21], shrimp shells [22], and mushrooms [23], which involve physical (CO_2_, H_2_O or O_2_) or chemical activation (H_3_PO_4_, HCl, ZnCl_2_, FeCl_3,_ KOH or NaOH) methods to produce tunable carbon materials, as well as allowing for the revalorization of waste [24,25]. However, having a low-value feedstock and using inexpensive chemicals does not guarantee that the valorization process is cheap. In most of these studies, a rigorous evaluation of costs is lacking. Likewise, even the obvious environmental friendliness could be questioned, taking into account the generation of undesirable compounds or new wastes (polluted wastewater) during the synthesis process of these biomass-derived carbons. These synthesis processes are not usually simple, but involve multiple and long stages, for example, the use of several-stage carbonization and activation methods, high porogen/precursor mass ratio, high carbonization temperatures (above 800 °C), slow rates and long times for pyrolysis steps, or the need for strong acid treatments for ultimate purification.

In particular, banana peels are a type of agricultural waste that make up of 30–40% of the total weight of the fruit, which presents a global production estimated at nearly 117 million tons in 2019 [26]. Nevertheless, the lack of economic value of this residue poses a major problem for food processing plants, since only a small part is intended as feedstock for animals or compost to fertilize farmland, while large quantities are discarded in landfills, where they are placed on garbage dumps and subsequently incinerated, releasing CO_2_ and harmful gases into the atmosphere [27]. Therefore, this not only represents a disposal problem, but also results in a great waste of natural energy resources due to the lack of adequate recycling processes [28,29].

Added value can be given to banana peels as raw materials to obtain activated carbons according to several precedents. Firstly, banana peels are composed of a fiber structure that includes biopolymers, such as lignin, cellulose, and hemicellulose, which contains hydrocarbon chains that form cross-linked and hierarchical frameworks. Secondly, lignin and hemicellulose content are useful for obtaining non-graphitic porous carbons at certain pyrolysis temperatures (500–1000 °C). During pyrolysis, volatile organic molecules are eliminated, as CO_2_, CO, H_2,_ H_2_O and the hydrocarbon chains are dehydrated, forming aromatic and disorderly stacked structures [30]. The use of chemical activation methods is advantageous because different porogens promote dehydration, aromatization and porosity formation with increasing temperature (400–900 °C), which is more effective in the development of carbonaceous structures with large specific surface areas and highly porous system [31,32]. Thus, this method allows for the development of activated carbons with controllable porosity and functionalized surfaces, necessary to make efficient and sustainable electrodes to enhance the electrochemical reactions in LIBs for the high-energy demands in renewable energy systems and electric vehicles [33].

**Scheme 1:**
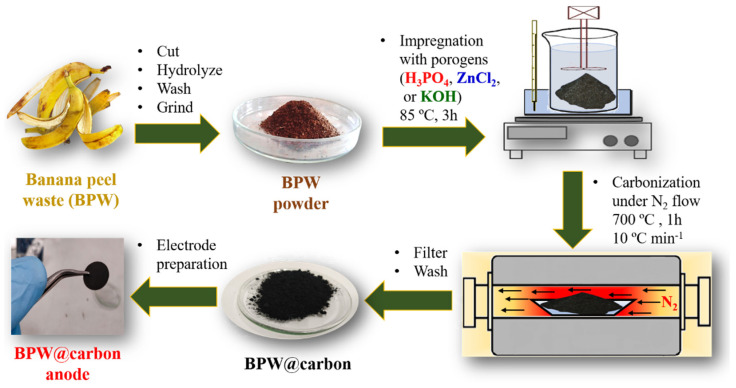
Illustration of synthesis process for BPW carbon anodes.

In this study, we propose a successful strategy to prepare added value porous carbons from banana peel waste (BPW) as a biomass precursor (Scheme 1). The employment of a one-step activation/carbonization method with different porogens allows us to develop porous and functionalized structures which, as a result of their multiple cavities and active sites, facilitate the accessibility of the electrolyte and improve the ion transfer kinetics. The electrochemical tests carried out of these biomass carbons as anodes in LIBs show good reversibility, capacity retention and excellent rate performance. This work illustrates the potential of these materials, compared with other biomass electrodes, through the use of a simple process towards the obtention of sustainable anodes for LIBs.

## 2. Materials and Methods

### 2.1. Materials

The BPW for this work was of the Cavendish variety from the Canary Islands (*Musa acuminate Colla AAA*), purchased from several markets in Córdoba, Spain. The chemical reagents for the synthesis of the activated carbons were of high purity and were supplied by different providers: sulfuric acid 96% (Panreac); zinc chloride 97% (Panreac); potassium hydroxide 85% (Panreac); and orthophosphoric acid 85% (Panreac). The chemical reagents for the preparation of the battery electrodes were of high purity and were supplied by different providers: poly(vinylidene fluoride) (PVDF) (Fluka); carbon black Super P (Timcal); 1-methyl-2-pyrrolidone anhydrous 99.5% (Sigma-Aldrich, St. Louis, MO, USA); and lithium hexafluorophosphate solution in ethylene carbonate and diethyl carbonate (1 M LiPF_6_ in EC/DEC (1:1; *v*/*v*), battery grade) (Sigma-Aldrich).

### 2.2. Activated Carbon Synthesis

The preparation of BPW activated carbons was carried out by employing a simple and sustainable method. Firstly, banana peels were cut into small pieces (2–3 cm), treated with a 5% (*v*/*v*) H_2_SO_4_ dilute solution under stirring at 25 °C for 3 h in order to initiate the catalytic hydrolysis of cellulose and hemicellulose, leaving only lignin units. Pieces were then washed with distilled water until neutral, before drying at 120 °C for 12 h. The dried residues were ground in a ball mill (Restch PM100, Retsch GmbH, Haan, Germany) at 300 rpm for 30 min, employing a 125 mL reaction recipient with eight stainless steel balls (diameter of 10 mm). Subsequently, the powder was impregnated with solutions of different activating agents/porogens (ZnCl_2_, H_3_PO_4_ and KOH) in a banana peel:porogen mass ratio of 1 and maintained under stirring and heating at 85 °C for 3 h until a solid paste was obtained, which was dried at 120 °C for 12 h. Finally, the impregnated products were heated in a quartz tubular oven (Carbolite Gero Ltd., Derbyshire, United Kingdom) up to 700 °C (10 °C min^−1^) and held for 1 h under a N_2_ atmosphere (flow rate of 50 mL min^−1^) to obtain the different activated carbons. After carbonization, successive activated carbons were filtered and washed with distilled water until neutral in order to remove most of the side products resulting from the dehydration and aromatization reactions that involve activating agents, in addition to the remaining mineral impurity traces from the raw material. To facilitate comparison, the carbons obtained with acidic, basic and zinc salt activation are labelled in the discussion as BPW@H_3_PO_4_, BPW@KOH and BPW@ZnCl_2_, respectively.

### 2.3. Characterization

The structure of the activated carbons was analyzed by X-ray diffraction (XRD) analysis with a D8 Discover (Bruker, Berlin, Germany) diffractometer with monochromatic Cu-K_α_ radiation (λ = 1.5406 Å). The diffractograms were recorded between 10° and 80° (2θ) with a step size of 0.02° and 0.2 s per step. Raman spectra were measured with a confocal Raman spectrometer ((alpha500, WITec, Ulm, Germany) with a frequency doubled Nd:YAG laser (532 nm). The laser beam was focused on the sample using a 20×/0.4 Zeiss objective, and the Raman spectra were recorded with an integration time of 1 s by accumulating a total of 10 spectra. The morphology characteristics of BPW carbons were obtained with a JEOL JSM-7800F scanning electron microscope (JEOL, Tokyo, Japan) equipped with an X-ACT Cambridge Instrument detector (Oxford Instruments, Abingdon, UK) for EDS elemental microanalysis. Samples were prepared by dispersing activated carbon powders on ethanol and depositing some drops of the suspension onto conductive carbon tape, used to adhesively bond the sample to the SEM cylinder-stub. The elemental analysis of the carbon and nitrogen content was carried out using a LECO CHNS–932 microanalyzer (LECO, Haan, Germany) by combustion at 1000 °C. The weight loss was determined by thermogravimetric analysis (TGA) with a Mettler Toledo-TGA/DSC under different atmospheres (oxygen and nitrogen, with a flux of 100 mL min^−1^) heating up from 30 to 800 °C at 10 °C min^−1^. The Brunauer–Emmett–Teller (BET) specific surface area was obtained from N_2_ adsorption-desorption isotherms at the temperature of nitrogen liquid (77 K) with a Micromeritics ASAP 2020, using nitrogen gas as an adsorbate. The pore size distribution was calculated using density functional theory (DFT).

### 2.4. Preparation of Electrodes

The electrodes for the LIBs were prepared by mixing several BPW activated carbons with the PVDF binder and carbon black Super P conductive agent in a mass proportion of 80:10:10 in 2.5 mL of 1-methyl-2-pyrrolidinone solvent. The slurries generated were coated onto a copper foil (9 μm in thickness) using the “doctor blade” technique (25 μm in thickness). Finally, the electrodes were cut on disks (13 mm in diameter) with a Gelon Group GN-T06 manual punching machine (Gelon Lib, Qingdao, China) and were dried in a glass oven (Buchi, Flawil, Switzerland) at 120 °C for 12 h until cell assembly. The active material loading in all anodes prepared for electrochemical tests was 1 mg cm^−2^.

### 2.5. Cell Assembly and Electrochemical Measurements

The cell assembly was performed on CR2032 coin cells using an argon-filled glove box (M-Braun 150 model; H_2_O ≤ 1 ppm) (M-Braun, Garching, Germany) with a lithium metal disk as the counter and reference electrodes (13 mm in diameter), and adding 30 μL of LiPF_6_ in EC:DEC (50:50; *v/v*) commercial electrolyte per anode. The electrochemical cycling tests were performed on a battery tester system (Arbin BT2143) in a voltage range of 0.01–3.0 V. For the galvanostatic discharge/charge tests, a constant current of 0.2 C (74.4 mA g^−1^) (considering the theoretical capacity of graphite 1 C = 372 mA g^−1^) was used. The rate capability tests were carried out at different current densities each for 10 cycles of 0.1 C, 0.2 C, 0.5 C, 0.8 C, 1 C, 2 C and returning to 0.1 C. The cyclic voltammetry (CV) and electrochemical impedance spectroscopy (EIS) measurements were recorded with a potentiostat Pgstat204 (Metrohm Autolab, Herisau, Switzerland). The CV measurements were performed at 0.1 mV s^−1^ between 0 and 3 V, and the EIS measurements were performed before and after the CV in a frequency range from 0.1 Hz to 500 kHz with a disturbance amplitude of 10 mV.

## 3. Results and Discussion

### 3.1. Structural, Chemical, and Textural Characterization

**Figure 1:**
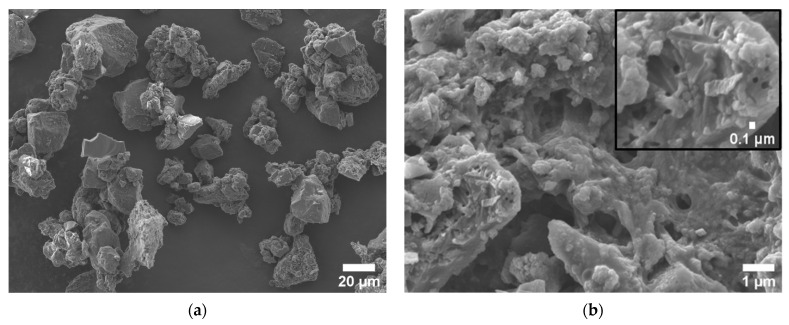

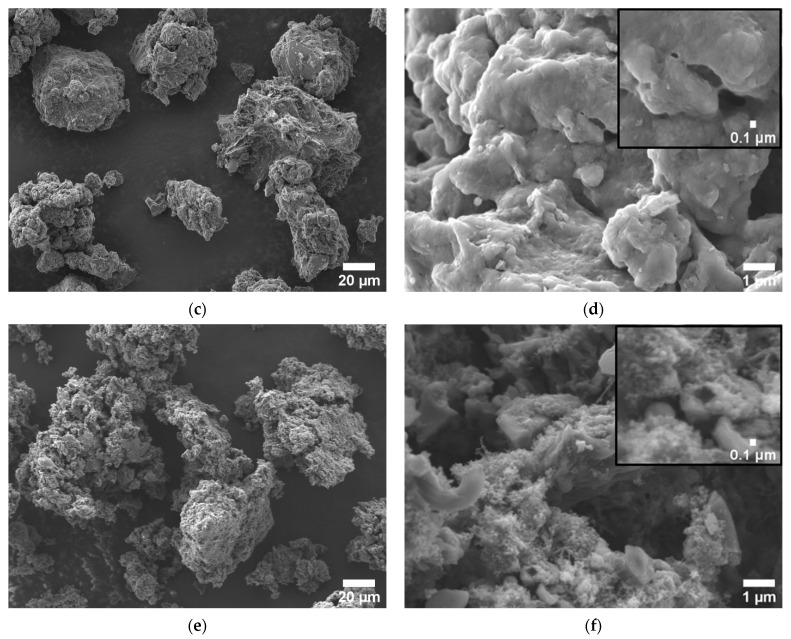
SEM images with different magnification of (**a**,**b**) BPW@H_3_PO_4_, (**c**,**d**) BPW@ZnCl_2_, and (**e**,**f**) BPW@KOH.

The morphology and structure of BPW-derived carbons investigated by SEM analysis are shown in Figure 1. In general, all samples show a tendency to form small aggregates of particles in the range of tens of micrometers which are composed of porous structures with irregular and complex morphologies, according to the porogen used in the carbonization of banana peels. The morphology of BPW@H_3_PO_4_ carbon (Figure 1a) is arranged as small particle blocks of different agglomeration degree and size (average size of 40 µm), which present rough surface and, in some cases, irregular polygonal shapes. At a higher magnification (Figure 1b), it can be determined that the structure of these particles is highly porous with the presence of many randomly oriented macropores and mesopores, which present different diameters and shapes (slit, cylindrical or spherical) (Figure 1b inset). Meanwhile, the morphology of BPW@ZnCl_2_ particles (Figure 1c) is also in the form of small aggregates of a higher degree (average size of 50 µm), although these present a rougher and more tortuous surface and rounded shapes which are poorly defined in some particles. The observation of a less developed porosity is evident (Figure 1d), with the presence of a large number of macropores but with few mesopores (slit-shape pores) (Figure 1d inset). Finally, BPW@KOH particles (Figure 1e) present a morphology in large aggregates (average size of 80 µm) which are completely amorphous and exhibit a characteristic sponge-like surface, typical in the activation with this porogen. Nevertheless, its surface hardly presents any small-sized porosity (Figure 1f), except for visible macropores in the structure (Figure 1f inset). The external porous framework of these carbons offers a high contact area that improves the preliminary impregnation/penetration of the electrolyte and lithium ions through its interconnected microchannels [34]. Meanwhile, the amorphous structure of their particles provides active sites and disordered regions to facilitate the adsorption and intercalation of lithium ions [35]. Furthermore, the presence of a notable quantity of macropores and mesopores inside, especially the majority which occur in BPW@H_3_PO_4_ carbon and a less relevant amount occurring in BPW@ZnCl_2_ and BPW@KOH carbons, can act as ion-buffering reservoirs and shorten the lithium ions diffusion pathways on the electrode surface [36]. It is important to note that the presence of visible micropores in these carbons could not be detected due to the difficulty in observing them in their complex structures with the power of resolution of the conventional SEM technique in order to obtain quality images at very high magnification. Thus, these morphological and structural features of BPW carbons are suitable for lithium storage applications.

**Figure 2:**
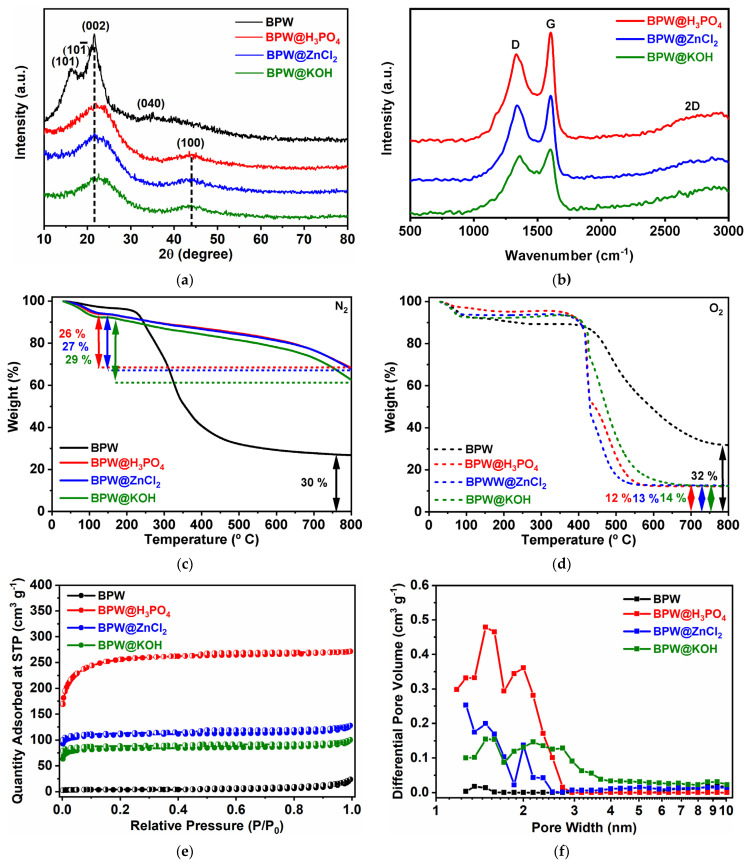
(**a**) XRD patterns of BPW raw material and successive activated carbons. (**b**) Raman spectra of raw material and activated carbons. (**c**) N_2_ (**d**) O_2_ TGA curves of BPW raw material and activated carbons. (**e**) Nitrogen adsorption-desorption isotherms of raw material and activated carbons. (**f**) DFT pore size distribution of BPW raw material and activated carbons.

Figure 2a shows the XRD patterns, with all BPW-derived carbons presenting the same two wide peaks at 23° and 43°, characteristic of the (002) and (100) reflection planes of graphite (PDF 41-1487), respectively, indicating the formation of disordered and amorphous carbonaceous structures. The weak (100) plane is related to the hexagonal arrangement, like a “honeycomb”, of sp^2^ hybridized carbon atoms forming graphene sheets [21], and the broad (002) plane suggests small irregularly ordered islands formed by logical and parallel stacking of graphene sheets [37]. Regarding the raw material, the diffractogram shows four peaks, three of them narrow and intense at 16°, 18° and 21°, and another broad peak and a few intense peaks at 35°, all of them from the natural cellulose crystallographic planes (101), (10-1), (002) and (040), respectively (PDF 03-0289) [38]. The natural cellulose content in the banana peels presents low crystallinity due to the presence of amorphous lignin in the fiber structures [39].

The different parameters extracted from the XRD analysis are shown in Table 1. Firstly, the interlayer spacing (d_002_) that is calculated from the center of the (002) peak using the Bragg equation (Equation (1)), where *λ* is the wavelength of the X-ray, *d* is the interlayer spacing, *θ* is the scattering angle, and *n* is an integer, shows that the activated carbons present larger distances between the graphene layers than graphite (d_002_ = 0.3354 nm), because of incrusting of the larger porogens inside, which facilitates the insertion/extraction of lithium ions and thereby increases the conductivity [40]. The Scherrer equation (Equation (2)), where *K* is the shape factor, *λ* is the wavelength of the X-ray, *θ* is the Bragg angle, and *B* is the FWHM of the (002) and (100) reflections, can be used to calculate *L* that is the mean crystallite size, for which the average thickness (L_c_) and width (L_a_) of the graphitic domains can be obtained. In the activated carbons, the average thickness oscillates between 1.7 and 1.8 nm and the width between 4.8 and 4.9 nm, indicating that all are stacked by four to five graphene layers according to the N parameter (L_c_/d_002_) [41]. Finally, the R factor, defined by Liu et al., is obtained by measuring the ratio between the (002) peak intensity (B) and the background intensity (A) [42]. The low values of R between 1.9 and 2.2 suggest that when R is lower, there is a small order degree with a high concentration of non-parallel single graphene layers, which is related to the higher specific capacity in LIBs [20]. Figure 2b shows the Raman spectra of the activated carbons with two characteristic peaks located at ~1343 cm^−1^ and ~1589 cm^−1^, which correspond to the D-band and G-band, respectively. The D band is ascribed to the defects and disorders in the carbon structure and the G is related to the sp^2^ hybridized carbons of the graphitic layers [41]. From the relative intensity ratio of the D-band to the G-band (I_D_/I_G_), the disorder degree (graphitization degree) of a carbon material can be determined [33]. As shown in Table 1, the I_D_/I_G_ values for BPW@H_3_PO_4_, BPW@ZnCl_2_ and BPW@KOH are 0.83, 0.87 and 0.90, respectively, indicating that these carbons are generally disordered and with defective structures (low graphitization degree). According to these values, it is worth mentioning the notable intensity of D-band in the Raman spectra of all carbons indicates the presence of a high content of defects and disordered regions, which promotes the insertion and storage of lithium ions into the carbon structure. Meanwhile, the higher intensity of the G-band over the D-band (lower value of I_D_/I_G_ ratio), which is superior in the BPW@H_3_PO_4_ carbon with respect to BPW@ZnCl_2_ and BPW@KOH carbons, corresponds to the presence of small size sp^2^ graphitic domains which enhance the electronic conductivity of the carbon electrode in lithium batteries [34,43].

(1) n·λ=2d·sinθ

(2) $$B_{size}\left(2\theta \right)=\frac{K\cdot \lambda }{L\cdot \cos\theta }$$

The TGA curves in the presence of N_2_ and O_2_ for all BPW samples are shown in Figure 2c,d, respectively. The TGA curves in N_2_ provide the carbonization temperature of BPW for the posterior preparation of several activated carbons. First, there is a weight loss of 4% related to the water evaporation and elimination of organic volatile compounds until 200 °C. The second weight loss of 70% between 200 and 500 °C involves the degradation of cross-linked cellulose and lignin units, starting the pyrolytic process of the formation of aromatic carbonaceous structures, with 30% of the carbon framework remaining. Meanwhile, the N_2_ TGA curves of the BPW-derived carbons presents an initial loss of weight of 6–8% by the water adsorbed in the porous structures and a significant weight loss between 26% and 29% is related to the decomposition of the thermolabile functional groups from 150 to 800 °C [44]. The TGA curves in the presence of O_2_ were recorded in order to determine the thermal stability. For activated carbons, the first weight loss of 4–8% between 100 and 150 °C is due to the physiosorbed water and the major second loss of 80–82%, ranging from 350 to 700 °C, is associated with the combustion of carbon in the samples, where the remaining 12–14% corresponds principally to the activating agent residues and, in a minor proportion, to the BPW natural ash traces. In the BPW raw material, a weight loss of moisture of 8% at 150 °C and a second weight loss of 57% from 330 to 800 °C associated with carbon elimination also appear, with 32% of the natural ash content remaining.

The elemental carbon and nitrogen content analysis (in mass%) is shown in Table 1 in order to quantify the purity of the samples. All BPW activated carbons presented a high content of carbon, exceeding 80%, which is consistent with the values obtained by TGA in O_2_ (Figure 2d) and EDS composition (Table S1). The elemental mapping images (Figures S1–S3) of three BPW samples demonstrate a homogeneous distribution of carbon and oxygen elements in their particles. The elevated percentages of carbon demonstrate the effectiveness in the formation of carbonaceous structures during pyrolysis. In the raw material, there is an expected minor content of C due to the major presence of O in the carbohydrate units and original ash content. Regarding the nitrogen (Table 1) and oxygen contents (Table S1), these are also remarkable in the BPW-derived carbons with percentages ranging from 1.2% to 1.75% and 6% to 11%, respectively. Nevertheless, the nitrogen signal by EDS analysis is almost undetectable and, therefore, neither the values of composition nor mapping images of this element are shown. The nitrogen presence comes from the protein units and the oxygen from the polysaccharides of the fiber structure of banana peels, whose higher initial values decrease in the respective carbons due to the loss of these biomolecules as volatile compounds during the activation and carbonization processes. It is noteworthy that in that in the compositions and mapping images of EDS analysis (Table S1 and Figures S1–S3), residual contents of respective porogens were detected, which are uniformly distributed in their structures. The coexistence of all of these elements can synergistically contribute to the formation of acidic (carboxylic, carbonyl and hydroxyl) and basic (pyrrolic and pyridinic) functional groups by heteroatoms (O and N), in addition to defects, channels and disordered regions in the carbon surface, by activating agents (P, Zn and K) which can act as effective active centers for the diffusion and storage of lithium ions into the carbon structure [45,46]. The significant content of natural silicon in all BPW samples can be explained by its natural precedence in some agricultural waste and the fact that its complete elimination is difficult. However, those silicon atoms that can occupy interstitial positions in the carbon do not seem to affect negatively the electrochemical performance, but rather improve the capacity and cell cycling in LIBs [47].

The textural properties were assessed by the N_2_ adsorption-desorption isotherms shown in Figure 2e. According to the IUPAC classification, the shape of the carbons isotherms is mixed type I/IV, where the first part indicates the presence of a micropore system at low relative pressures (P/P_0_ < 0.4) and the second part the presence of a H4 hysteresis loop at high relative pressures (P/P_0_ = 0.4–1), related to the phenomenon of capillary condensation in mesopores [48]. Thus, these activated carbons present a slit-shaped pore structure, where there is a dominant presence of micropores and a significant proportion of mesopores. As expected, the raw material shows a type-II isotherm due to the lack of porosity in the bulk material. The specific surface area values based on the BET method are recollected in Table 1, where the BPW@H_3_PO_4_ carbon presents the highest S_BET_ of 815 m^2^ g^−1^, principally given by adsorption in micropores, whilst the BPW@ZnCl_2_ and BPW@KOH carbons present lower values of S_BET_ with 348 and 264 m^2^ g^−1^, respectively.

Figure 2f shows the pore size distribution obtained by the non-local DFT model for porous carbonaceous materials [49], according to which these activated carbons present a high concentration of micropores in the region of 1–2 nm and a lower proportion of mesopores at 3–10 nm. From the volume and pore size values extracted from DFT (Table 1), the BPW@H_3_PO_4_ carbon presents the highest pore volume of 0.42 cm^3^ g^−1^, given by the predominant microporous system, which is in contrast with its low average pore size of 2.08 nm which is slightly above the microporous region. This one-step carbonization/activation procedure of BPW, especially with the H_3_PO_4_ porogen, allows for the development of disordered and functionalized carbonaceous structures with large specific surface area and hierarchical porosity, composed of micropores, mesopores, reactive functional groups and interstitial defects. First, the distribution of pores provides pathways that promote the impregnation of the electrolyte and facilitate the diffusion/insertion of lithium ions into the carbon [50]. Second, the defects and surface oxygen and nitrogen groups serve as active sites, thereby promoting the transfer and trapping of more lithium ions [51]. Thus, these activated carbons have excellent physical and chemical properties to be tested in LIBs.

### 3.2. Electrochemical Properties in LIBs

**Figure 3:**
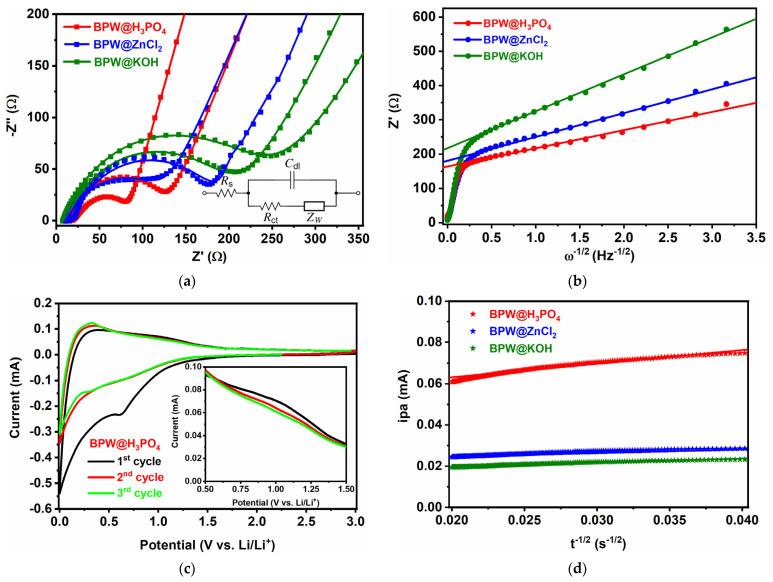
(**a**) Fitted Nyquist plots of electrodes before and after CV. (Inset) Equivalent circuit model used. (**b**) Linear fitting of Warburg impedance of electrodes. (**c**) CV curves recorded at 0.1 mV s^−1^ of BPW@H_3_PO_4_ electrode. (Inset) Zoom of ‘‘hump’’ region. (**d**) Fitted response of current versus t^−1/2^ for electrodes with the half discharge degree under the ‘‘hump’’ potential.

The electrochemical performance of the BPW electrodes was studied using lithium-ion half-cell configurations as anodes versus Li metal as the counter electrode. To determine the resistance of BPW-derived carbon electrodes, EIS was carried out in a frequency range from 0.1 Hz to 500 kHz, before and after CV. As shown in Figure 3a, the Nyquist plots are composed of a single depressed semicircle in the high-to-medium frequency region and an inclined tail in the low frequency region. The semicircle is associated with the combined effects of the contact resistance between interfaces, electrode resistance and solid electrolyte interphase (SEI) resistance, given by R_e_, in addition to the transfer charge resistance at the electrode/electrolyte interface, given by R_ct_, while the tail (Warburg impedance), given by Z_w_, is attributed to the diffusion of lithium ions into the carbon electrode. Data fitting was performed by simulation with a Randles equivalent circuit model (Figure 2a inset), where R_e_ indicates the electrolyte resistance, R_ct_ the charge transfer resistance, CPE (C_dl_) the double layer capacitance and Z_w_ the diffusion of lithium ions through the carbon electrode [52].

Before CV, the R_ct_ values were 114, 172 and 234 Ω for the BPW@H_3_PO_4_, BPW@ZnCl_2_ and BPW@KOH electrodes, respectively. After CV, the semicircles were reduced and the R_ct_ values were notably decreased to 63, 119 and 184 Ω, indicating a lower resistance due to the good electrochemical response of the electrodes. It also should be noted that the R_e_ values for different electrodes are lower after CV, due to a decrease in electrolyte resistance with the charge-discharge reactions [50]. Furthermore, the Warburg impedance Z_w_ slopes of different electrodes are larger after than before CV, which reflects the better diffusion of lithium ions during cycling [43]. The Warburg coefficients (A_w_) can be extracted from the slope of the Z’ vs. ω^−1/2^ line at low frequency in Figure 3b, where ω is the angular frequency of the alternating current. In the equation reported by Zhang et al., the Li-ion diffusion is directly proportional to Z_w_ and (1/A_w_)^2^ [53]. The A_w_ coefficients extracted for BPW@H_3_PO_4,_ BPW@ZnCl_2_ and BPW@KOH are 50, 68 and 107 Ω s^−1/2^, respectively, confirming that the lower slope determines higher ionic conductivity [34]. From the Warburg region, coefficients for Li^+^ diffusion (D_Li_^+^_EIS_) which transferred from the electrolyte into the carbon electrode interface can be obtained according to the formula based on previous model proposed by Ho et al., where R is the gas constant, T is the absolute temperature, A is the electrode surface area, n is the number of electrons per molecule involved in the redox process, F is the Faraday constant, C is the concentration of Li^+^ (shuttle concentration), and σ_w_ is the Warburg factor from the slope coefficients (A_w_), calculated above [54]. BPW@H_3_PO_4_ anode presents the smallest R_e_, R_ct_ and A_w_, which indicates good electronic conductivity reflected by a higher Li^+^ diffusion coefficient of 7.94 × 10^−12^ cm^2^ s^−1^ than BPW@ZnCl_2_ and BPW@KOH, which differs slightly with values of 4.24 × 10^−12^ cm^2^ s^−1^ and 1.74 × 10^−12^ cm^2^ s^−1^, respectively (Table S2). This is derived from a large porous structure and high specific surface area that reduce the transmission distance of lithium ions and also offer a suitable electrode/electrolyte interface to facilitate the transport of lithium ions into the carbon electrode, thereby improving the cycling performance [36].

In order to determine the electrochemical process in the carbon electrode, the CV curves of the BPW@H_3_PO_4_ anode are represented in Figure 3c within a voltage range of 0.01 to 3.0 V and under a scan rate of 0.1 mV s^−1^. This material shows typical CV curves for hard carbons, where the first cycle differs considerably from the subsequent cycles. Firstly, in the reduction step of the 1st cycle, a weak cathodic peak at 0.7 V appears, associated with the electrolyte decomposition on the surface induced by the formation of the SEI layer and also due to the irreversible insertion of lithium ions into special positions in the carbon structure [55,56]. This peak does not appear in the second and third cycles, which indicates the good reversibility of this electrode during the insertion/extraction of lithium ions. Between 0.01 and 0.5 V, a sharp reduction peak appears, which is attributed to the diffusion paths of lithium ions during the intercalation process on the carbonaceous structure [57]. In the oxidation step, a broad anodic peak appears between 0.1 and 0.4 V, which is assigned to the contrary deintercalation process of lithium ions from the stacked layers of the carbonaceous porous structure [58]. Furthermore, the appearance of a “hump” between 0.5 and 1.2 V is commonly ascribed to the adsorption/desorption processes of lithium ions from the porous carbonaceous structure [59]. The prevalence between the intercalation/deintercalation and adsorption/desorption processes of lithium ions into the carbon electrodes depends on different factors, such as the graphitization degree, the porous structure, and the functional groups and defects, which are key to guaranteeing the reversibility and diffusion of lithium ions.

The second and third cycles nearly overlap, indicating high redox reversibility and good stability of lithium storage in the electrode. Furthermore, it is observed that the plot area of BPW@H_3_PO_4_ is greater than BPW@ZnCl_2_ and BPW@KOH (Figure S4), due to a more developed porous structure which releases a higher specific capacity. According to the Li^+^ diffusion coefficients obtained from the Warburg region of EIS analysis, diffusion coefficients can also be calculated during the intercalation/deintercalation process of lithium ions into the porous carbons. This process occurs during the half-discharge step corresponding to the hump region from 0.5 to 1.2 V in the CV oxidation scan (Figure 3c and Figure S3 insets). Considering a series of previous hypotheses for carbon particles, the diffusion coefficients can be obtained using the equation deduced for carbon anode materials by Guo et al. [60]. Figure 3d shows that there is a linear relationship between the current (i) of the carbon electrode and time (t^−1/2^), where the intercept (b), slope (k) and radius of the particles (R_0_) can be used to extract the diffusion coefficient of Li^+^ (D_Li_^+^_CV_). BPW@H_3_PO_4_. A large specific surface area presents the highest value of Li^+^ diffusion with 8.23 × 10^−8^ cm^2^ s^−1^, while BPW@ZnCl_2_ and BPW@KOH present lower values with 3.76 × 10^−8^ and 1.99 × 10^−8^ cm^2^ s^−1^, respectively (Table S2). The values mentioned above differ by several magnitude orders compared to those obtained from the EIS diffusion processes. These results indicate that a larger porous structure offers more diffusion channels to facilitate the transport and increase the kinetic reactions of lithium ions into the carbon electrode [35].

**Figure 4:**
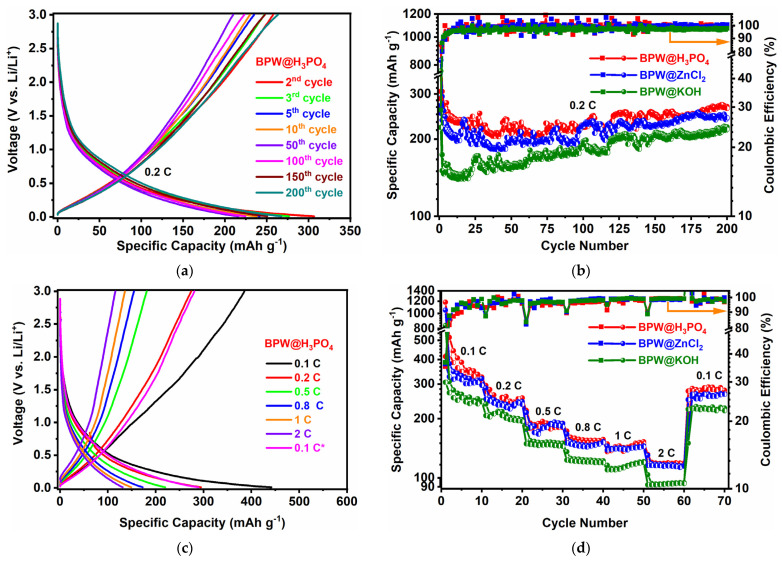
(**a**) Charge–discharge profiles at 0.2 C (74.4 mA g^−1^) of BPW@H_3_PO_4_ anode. (**b**) Specific capacity versus cycle number and coulombic efficiency of activated carbons at 0.2 C. (**c**) Charge–discharge profiles at different current rates of BPW@H_3_PO_4_ anode. (**d**) Rate capability tests of activated carbons at different current rates.

In order to evaluate the cycling performance, galvanostatic regime measurements and rate capability tests were carried out. Figure 4a shows the charge–discharge curves of the BPW@H_3_PO_4_ anode between 0.01 and 3 V at 0.2 C (74.4 mA g^−1^), while the BPW@ZnCl_2_ and BPW@KOH curves are illustrated in Figure S6. Initially, all carbons released high discharge capacities of 942, 831 and 763 mAh g^−1^ for BPW@H_3_PO_4_, BPW@ZnCl_2_ and BPW@KOH, respectively (Figure S5), with a marked plateau of 0.5–1 V, in line with the cathodic peak assigned to 0.7 V in the first CV curve. In contrast, the capacities exhibited in the first charge cycle were only 307, 253 and 222 mAh g^−1^, with low values of coulombic efficiency (CE) over 30%. This inevitable phenomenon is explained by the high degree of irreversible trapping of the Li ions of the external functional groups and porous cavities on the carbon, and also by the decomposition of the electrolyte and the subsequent SEI membrane formation on the electrode surface [19,61]. Nevertheless, from the 2nd to 200th cycle, the charge and discharge branches nearly overlap, with the CE above 98%, reflecting the good electrochemical reversibility and the full stability of the SEI layer reached by the porous carbon structure with cell cycling [62].

The cycling performance of the electrodes at 0.2 C in Figure 4b shows the good reversible capacities delivered for BPW@H_3_PO_4_, BPW@ZnCl_2_ and BPW@KOH after 200 cycles of 272, 250 and 224 mAh g^−1^, respectively. According to this figure, it should be noted that all electrodes present a slight ascendant tendency in the capacity values between 7% and 16% from the initial cycles until the 200th cycle, reaching a CE close to 100%. This behavior may be attributed to an unusual phenomenon that involves the original SEI film rupturing and the subsequent formation of a new and thinner SEI film, originating from an increase in the specific capacity [63]. Furthermore, there are considerable differences in the capacities of the carbon activated with H_3_PO_4_ compared with ZnCl_2_ or KOH, due to the larger specific surface area and pore volume already discussed. This means a higher porosity, providing more vacancies to insert more Li ions inside and increasing the transfer kinetics of the redox reactions in LIBs [64].

Figure 4c,d display the charge–discharge curves for the BPW@H_3_PO_4_ anode, and the rate capability tests, respectively, at current rates of 0.1 C, 0.2 C, 0.5 C, 0.8 C, 1 C, 2 C and 0.1 C. According to the tests, the BPW@H_3_PO_4_ electrode exhibited the highest discharge capacities of 442, 295, 220, 173, 149 and 131 mAh g^−1^ at 0.1 C, 0.2 C, 0.5 C, 0.8 C, 1 C and 2 C, respectively. Finally, the discharge capacity recovered notably with 284 mAh g^−1^ when the current returned to 0.1 C, showing a good capacity for recovery. Regarding the other carbons, their rate performances, and respective curves (Figure S6) showed lower capacity values, which become more evident at low current rates, as discussed above for the galvanostatic measurements. BPW@ZnCl_2_ showed good discharge capacities at the same current densities of 347, 286, 214, 167, 145, 129 and 267 mAh g^−1^, while BPW@KOH presented lower values of 280, 237, 178, 136, 116, 104 and 230 mAh g^−1^, respectively. The electrodes present a capacity fade in the first cycles, which could be the result of the longer electrochemical activation process required for full stable SEI layer formation in hard carbons and the marked cell polarization at high current densities [65,66]. Notable differences between the charge and discharge capacities in the first cycles are also observed, which may be caused by the irreversible insertion of Li ions into defects on disordered carbons [67]. In general, the BPW electrodes present a good rate performance with high reversibility of the charge–discharge reactions and excellent capacity retention, both for long cycling and at different current rates (Table S3).

The favorable electrochemical response exhibited by BPW anodes in lithium-ion batteries, especially in the BPW@H_3_PO_4_ sample, can be explained by several reasons closely related to their unique functional and porous structure analyzed by different techniques. Firstly, the microstructure of the amorphous carbon particles, formed by simultaneous carbonization and activation with different porogens, guarantees enough contact area for total electrolyte permeability, in addition to presenting many defects and disorders suitable for accessibility and the accumulation of charges into the electrode surface, thus reducing the electronic resistance between interfaces and the volumetric changes during the charge–discharge process. Secondly, the existence of a significant content of small graphitic domains in the disordered structures helps to improve the ion transfer kinetics, which translates into good electronic conductivity and excellent cycle stability. Thirdly, the interconnected porous regions of these particles are composed of macropores and can act as ion-buffering reservoirs, as well as mesopores and micropores which provide shorter pathways for better diffusion of lithium ions. Hence, the presence of this developed pore-system, predominantly microporous, gives a large surface area and amount of active sites which ensure the insertion/interfacial storage of more lithium ions into the structure, achieving a high initial irreversible capacity in addition to a good cycling and rate performance. Fourthly, the presence of many micropores also contributes to reaching a high reversible capacity, even at high current densities. Lastly, all of these key factors discussed above contribute synergistically to the optimal lithium storage in the BPW biomass carbon structure. [48,53,65,67,68].

In summary, BPW activated carbons have proven to be promising anodes for LIBs in terms of electrochemical reversibility and stability. For comparison, Table 2 shows several biomass-derived carbons that are reported in the literature, some of which have superior or similar cycling and rate performances, but often involve complex, wasteful or unsustainable procedures. These carbons were derived, for example, from the use of two-stage carbonization and activation methods [23,69], the employment of high carbonization temperatures (>800 °C) [15,63,70], the use of a high ratio of porogen quantity in relation to the precursor [71,72,73,74], the employment of slow rates or long times for pyrolysis [16,18,75,76], or the use of strong acid treatments for carbon purification [77]. All of these studies entail a waste of energetic and material resources, in addition to being less green and eco-friendly, meaning that they are of less interest from economic and commercial viewpoints for LIB manufacturing. In contrast, our BPW activated carbons were obtained using a simple and direct method following a one-step activation/carbonization method at 700 °C, with a 1:1 porogen/precursor ratio and employing short times, resources, and soft treatments. The BPW@H_3_PO_4_ carbon showed a developed and functional porous structure, with a high content of micropores and active sites, which provides a large specific surface area of 815 m^2^ g^−1^, comparable with other reported works [20,21]. From the electrochemical point of view, the major porosity gives the carbon a high initial and irreversible capacity of 921 mAh g^−1^ at 0.2 C, which is comparable to some carbons reported, such as cherry stones (900 mAh g^−1^ at 0.1 C), coconut shells (1714 mAh g^−1^ at 0.1 C), or sisal fibers (998 mAh g^−1^ at 0.1 C) [20,71,74]. In fact, this good porous structure also confers a positive cycling response, with a stable and reversible capacity of 272 mAh g^−1^ at 0.2 C, higher than other biomasses, among which include olive stones (170 mAh g^−1^ at 0.2 C), soybean curd (90 mAh g^−1^ at 0.2 C), or banana fibers (217 mAh g^−1^ at 0.1 C) [21,73,74], although lower than those reported by other studies even at inferior or similar current rates, such as walnut shell (380 mAh g^−1^ at 0.08 C), pinecone hull (394 mAh g^−1^ at 0.02 C), or coffee shells (300 mAh g^−1^ at 0.2 C) [63,69,72] In terms of cycling stability, this carbon supplied more than the specific capacity of up to 200 cycles, which demonstrates its excellent capacity retention which is better than that other biomasses, for instance tea leaves (63 mAh g^−1^ after 100 cycles at 0.1 C), sisal fibers (240 mAh g^−1^ after 30 cycles at 0.1 C), or soap-nut seeds (130 mAh g^−1^ after 100 cycles at 0.1 C) [16,76,77], even though other works reported comparable reversible capacities at higher cycle numbers, such as bamboo wood (170 mAh g^−1^ after 300 cycles at 1 C) or mushroom skin (260 mAh g^−1^ after 700 cycles at 0.27 C) [15,23]. Finally, the rate performance of this carbon is acceptable, delivering specific capacities of 149 and 131 mAh g^−1^ at 1 C and 2 C, respectively, although it is inferior the rate performance of some materials discussed in other works, including rice straw (165 mAh g^−1^ at 0.7 C), or wood pieces (150 mAh g^−1^ at 2 C) [18,70]. Good conductivity completes its remarkable potential to supply high energy in LIBs. However, these properties do not warrant the use of this material for commercial Li-ion battery anode. A high specific surface area causes a high first cycle loss and, hence, the overall battery capacity in a full cell will reduce significantly. This drawback could be converted to an advantage if high surface biomass derived carbon is used as an electroactive material in supercapacitors (EDLCs) or ion-capacitors (ICs) [78], or even as an anode for Na-ion batteries, where hard carbons are highly recommended [79].

## 4. Conclusions

In this work, several biomass carbons derived from banana peel waste were obtained by a simple and economical one-step synthesis procedure. The chemical activation was carried out with three different porogens (H_3_PO_4_, ZnCl_2_ and KOH) obtained through posterior pyrolysis at 700 °C for the respective activated carbons. These carbon-rich materials presented disorder, structural stability, and hierarchical porosity optimal for facilitating the insertion of lithium ions inside their structures. The BPW@H_3_PO_4_ carbon presented the largest specific surface area of 815 m^2^ g^−1^ given by a dual-pore system with the presence of micropores, making it a suitable material to be tested in LIBs. This anode exhibited the best electrochemical properties, achieving a reversible capacity of 272 mAh g^−1^ at 0.2 C after 200 cycles, in addition to presenting low resistance, good diffusion of lithium ions, electrochemical stability and excellent capacity retention, even at high current rates, showing it to be an anode candidate for high-energy LIBs. However, key drawbacks, such as irreversible high capacity, must still be overcome before considering BPW-derived carbons as possible commercial Li-ion battery anode. Likewise, a rigorous cost evaluation and comprehensive environmental impact assessment of the entire valorization process will be necessary prior to its large-scale study.

## Figures and Tables

**Table 1:** Composition, structure, and textural properties for BPW-derived carbons.

Sample	C (%) ^1^	N (%) ^1^	d_002_ (nm)	L_c_ (nm) ^2^	L_a_ (nm) ^3^	N Layers	R Factor	I_D_/I_G_ Ratio	S_BET_ (m^2^ g^−1^)	V_total_ (cm^3^ g^−1^)	V_micro_ (cm^3^ g^−1^)	Pore Size (nm)
BPW	49.9	2.45	-	-	-	-	-	-	12	0.036	0.004	12.5
BPW@H_3_PO_4_	86.25	1.75	0.393	1.85	4.91	4.71	1.96	0.83	815	0.420	0.332	2.08
BPW@ZnCl_2_	85.73	1.53	0.397	1.83	4.86	4.61	2.03	0.87	348	0.198	0.154	2.31
BPW@KOH	83.15	1.19	0.399	1.72	4.83	4.31	2.20	0.90	264	0.155	0.117	2.39

^1^ Weight (% mass). ^2^ L_c_ → *K* = 0.91. ^3^ L_a_ → *K* = 1.84.

**Table 2:** Comparison of electrochemical performance of BPW@H_3_PO_4_ electrode with other biomass-derived carbonaceous materials reported in the literature.

Biomass Precursor	Long-Term Cycling				Rate Capability			Reference
	Initial Capacity (mAh g^−1^)	Reversible Capacity (mAh g^−1^)	Cycles	Rate (C) ^1^	Average Capacity (mAh g^−1^)	Cycles	Rate (C) ^1^	
Soybean curd	102	90	5	0.2	40	5	1	[73]
					25	5	2	
Bamboo wood	169	170	300	1	82	3	1	[15]
					41	3	3	
Soap-nut seeds	650	130	100	0.1	-	-	-	[77]
Tea leaves	100	63	100	0.1	-	-	-	[16]
Rice straw	562	300	5	0.07	175	5	0.7	[18]
Sisal fibers	998	250	40	0.1	-	-	-	[75]
Sisal fibers	650	240	30	0.1	-	-	-	[76]
Mushroom skin	771 ^2^	260	700	0.27	-	-	-	[23]
Wood pieces	466 ^3^	231	200	1	243	5	1	[70]
					150	5	2	
Pinecone hull	660 ^4^	394	8	0.02	-	-	-	[69]
Coconut shells	1714	250	15	0.1	-	-	-	[71]
Olive stones	615	170	100	0.2	130	100	1	[21]
					105	100	2	
Cherry stones	900	287	100	0.1	278	100	1	[20]
					243	100	2	
Coffee shells	1200	300	15	0.2	-	-	-	[72]
Walnut shell	410	380	50	0.08	190	10	2.7	[63]
					180	10	5.4	
Banana fibers	921	217	10	0.1	-	-	-	[74]
Banana peels	942	272	200	0.2	149	10	1	This work
					131	10	2	

^1^ C rate is referred to the theoretical capacity of graphite (1 C = 372 mA g^−1^). ^2^ The first cycle was performed at 0.13 C (theoretical limit capacity: 1 C = 500 mAh g^−1^). ^3^ The first three cycles were performed at 0.1 C. ^4^ The first six cycles were performed at 0.03 C.

## Data Availability

The data presented in this study are available on request from the corresponding author.

## Supplementary Materials

The following are available online at https://www.mdpi.com/article/10.3390/ma14205995/s1.
