# Supplementary material for: Biomass Porous Carbons Derived from Banana Peel Waste as Sustainable Anodes for Lithium-Ion Batteries
Source: Materials (Basel). 2021 Oct 12;14(20):5995. doi: 10.3390/ma14205995 (PMC8538914; doi:10.3390/ma14205995)
Supplement: Supplementary file 1 [file materials-14-05995-s001.zip › materials-1338085-supplementary.pdf]

materials MDPI

Supporting information

Biomass porous carbons derived from banana peel waste as sustainable anodes for lithium-ion batteries

Fernando Luna-Lama, Julián Morales and Alvaro Caballero *

Dpto. Química Inorgánica e Ingeniería Química, Instituto Universitario de Química Fina y Nanoquímica (IUNAN), Facultad de Ciencias, Universidad de Córdoba, 14071 Córdoba, Spain; q12lulaf@uco.es (F.L.-L.); iq1mopaj@uco.es (J.M.)

* Correspondence: alvaro.caballero@uco.es; Tel.: +34 957218620

1. Elemental composition of the BPW samples obtained by EDS analysis.

Table S1. Elemental composition of the BPW samples obtained by EDS analysis.

Element [1]	*BPW*	*BPW@H_3PO_4*	*BPW@$ZnCl_2$*	*BPW@KOH*
C	52.98	89.91	87.26	83.01
O	37.57	7.01	6.51	11.57
Si	3.46	1.08	2.44	2.78
P	0.85	2.00	-	-
Zn	0.51	-	3.11	-
Cl	-	-	0.68	-
K	1.52	-	-	2.64
Mg	0.73	-	-	-
Ca	1.32	-	-	-
Na	0.56	-	-	-
Fe	0.24	-	-	-
Cu	0.15	-	-	-
S	0.11	-	-	-

[1] Weight (atomic %).

2. Kinetic parameters for carbon electrodes from fitted EIS and CV measures

Table S2. Kinetic parameters for carbon electrodes from fitted EIS and CV measures.

Sample	*$R_{e(initial)}$* (Ω)	*$R_{e(final)}$* (Ω)	*$R_{ct(initial)}$* (Ω)	*$R_{ct(final)}$* (Ω)	*A_w* ($Ω\ s^{-1/2}$)	*D_{Li^+EIS}* ($cm^2\ s^{-1}$) [1]	*D_{Li^+CV}* ($cm^2\ s^{-1}$) [2]
BPW@H_3PO_4	10.77	7.42	114	63.4	50.3570	7.94×10^{-12}	8.23×10^{-8}
BPW@$ZnCl_2$	14.93	11.09	172	117	68.9113	4.24×10^{-12}	3.76×10^{-8}
BPW@KOH	19.23	15.69	234	184	107.6097	1.74×10^{-12}	1.99×10^{-8}

[1] Calculated for an area of the electrode of 1.327 cm^2. [2] Calculated for an average particle radius of 25 μm.

3. Specific capacities of BPW electrodes at different cycles for 0.2 C galvanostatic regime and at different current densities for rate capability test

Table S3. Specific capacities (in mAh g^{-1}) of BPW electrodes at different cycles for 0.2 C galvanostatic regime and at different current densities for rate capability test.

Sample	***Cycle***	***1st***	***2nd***	***5th***	***10th***	***20th***	***50th***	***100th***	***150th***	***200th***
BPW@H_3PO_4		942	307	277	242	238	212	225	250	272
BPW@$ZnCl_2$		831	293	228	207	211	195	226	227	250
BPW@KOH		763	174	157	147	146	158	195	212	224

Sample	***Rate***	***0.1 C***	***0.2 C***	***0.5 C***	***0.8 C***	***1 C***	***2 C***	***0.1 C [1]***
BPW@H_3PO_4		442	295	220	173	149	131	284
BPW@$ZnCl_2$		347	286	214	167	145	129	267
BPW@KOH		280	237	178	136	116	104	230

[1] Return to 0.1 C in the rate capability test.

4. EDS spectra and elemental mappings of BPW@H_3PO_4, BPW@$ZnCl_2$, BPW@KOH

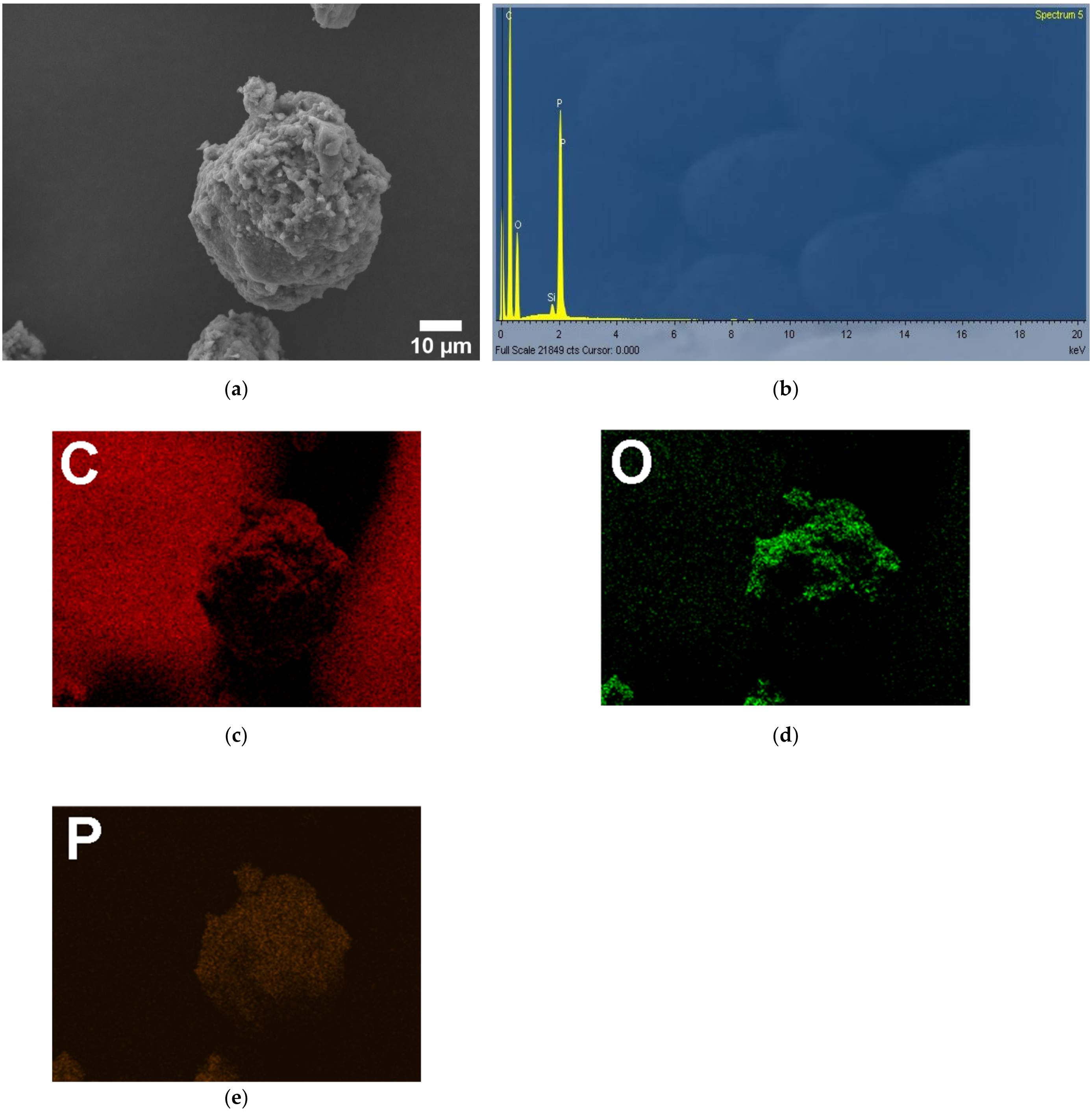

Figure S1. (**a**) EDS image, (**b**) EDS spectrum, (**c**) C, (**d**) O, and (**e**) P elemental mapping of BPW@H_3PO_4.

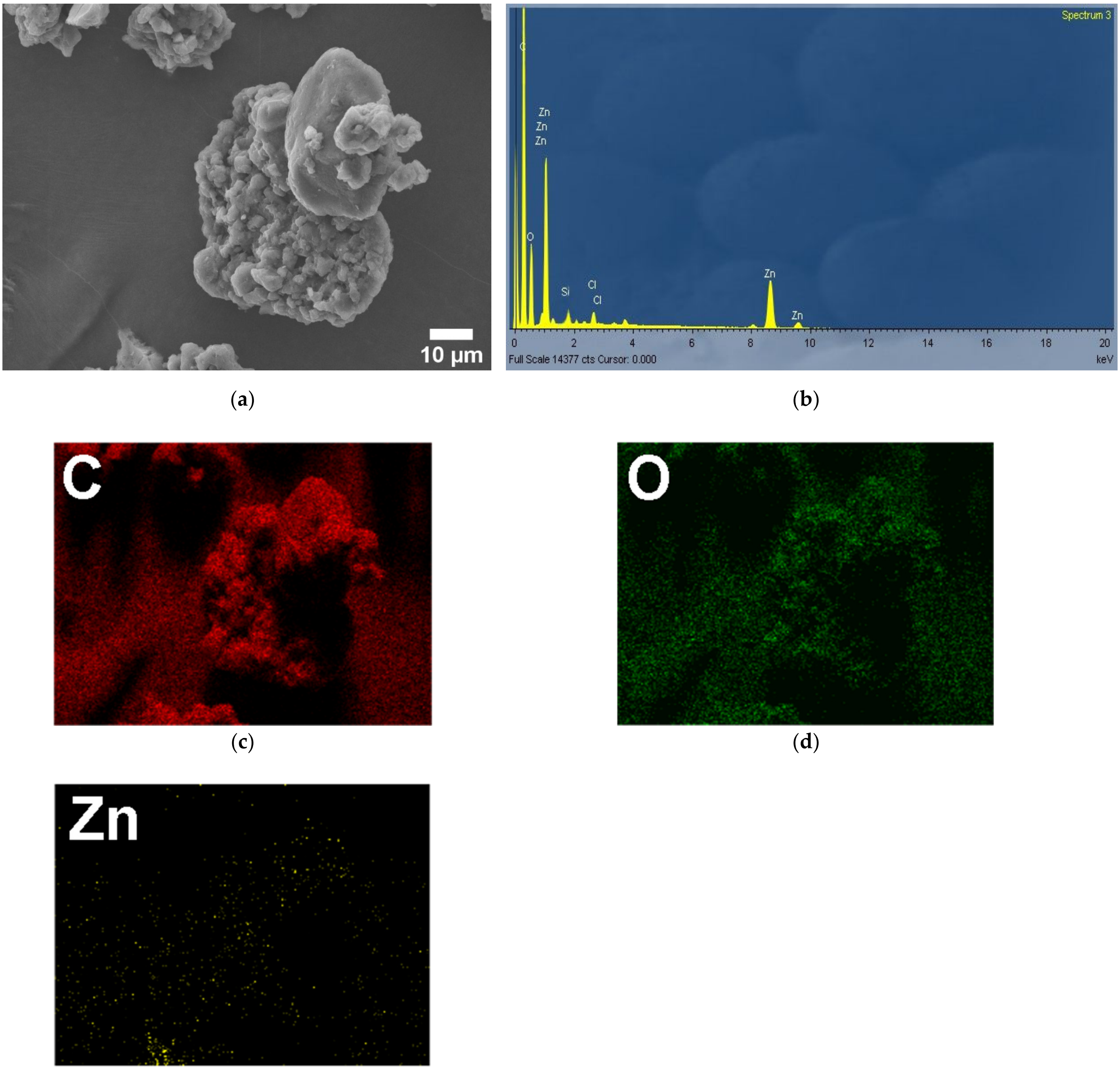

(**a**) (**b**)

(**c**) (**d**)

(**e**)

Figure S2. (**a**) EDS image, (**b**) EDS spectrum, (**c**) C, (**d**) O, and (**e**) P elemental mapping of BPW@$ZnCl_2$.

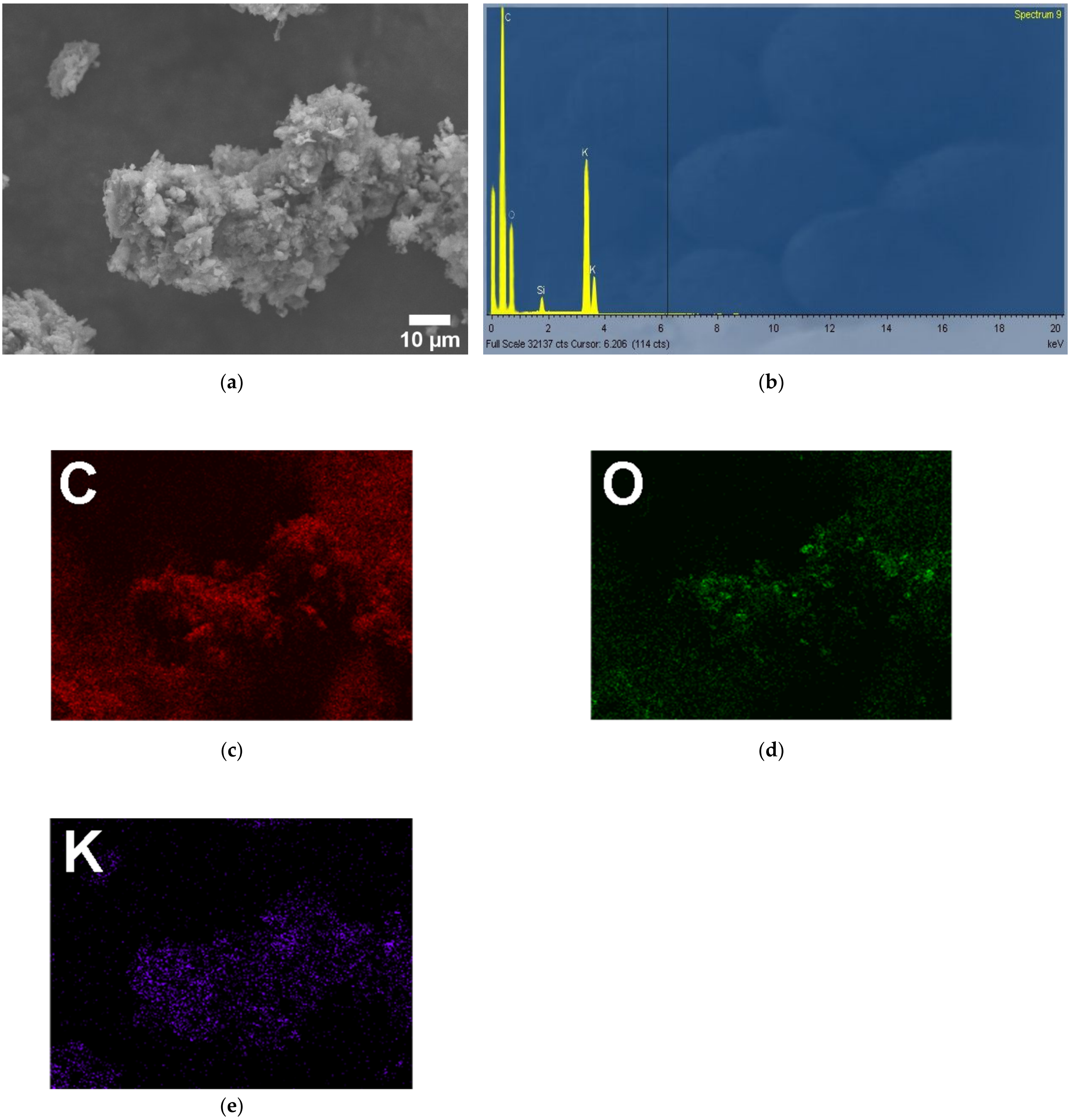

Figure S3. (**a**) EDS image, (**b**) EDS spectrum, (**c**) C, (**d**) O, and (**e**) P elemental mapping of BPW@KOH.

5. CV curves recorded at 0.1 mV s-1 of BPW@ZnCl2 and BPW@KOH

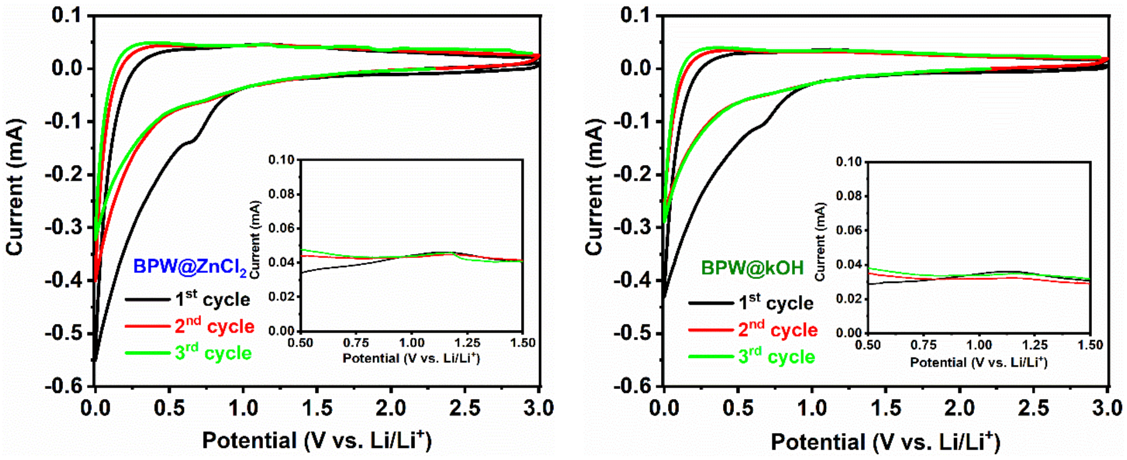

(a) (b)

Figure S4. CV curves recorded at 0.1 mV s-1 of (**a**) BPW@ZnCl2, (**b**) BPW@KOH. (Insets) Zoom of "hump" regions.

6. Charge-discharge curves of 1st cycle at 0.2C for BPW@H3PO4, BPW@ZnCl2 and BPW@KOH

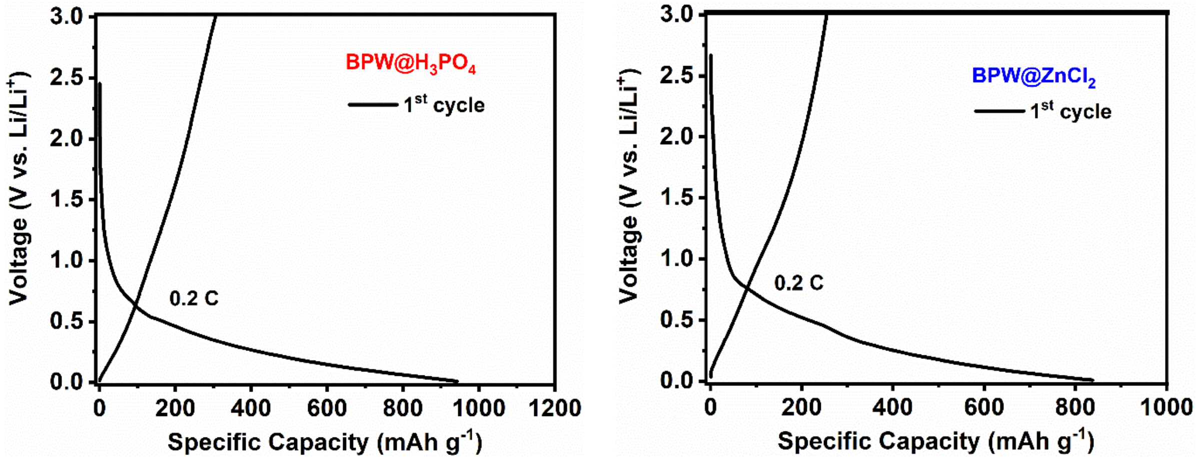

(a) (b)

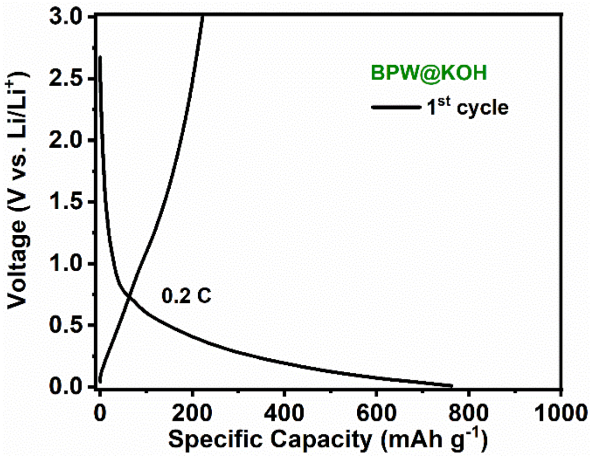

(c)

Figure S5. Galvanostatic charge-discharge curves of 1st cycle at 0.2 C for (**a**) BPW@H3PO4, (**b**) BPW@ZnCl2, (**c**) BPW@KOH

7. Galvanostatic charge-discharge curves from 2nd to 250th cycle at 0.2 C for BPW@ZnCl$_2$ and BPW@KOH. Rate capability charge-discharge curves for BPW@ZnCl$_2$ and BPW@KOH

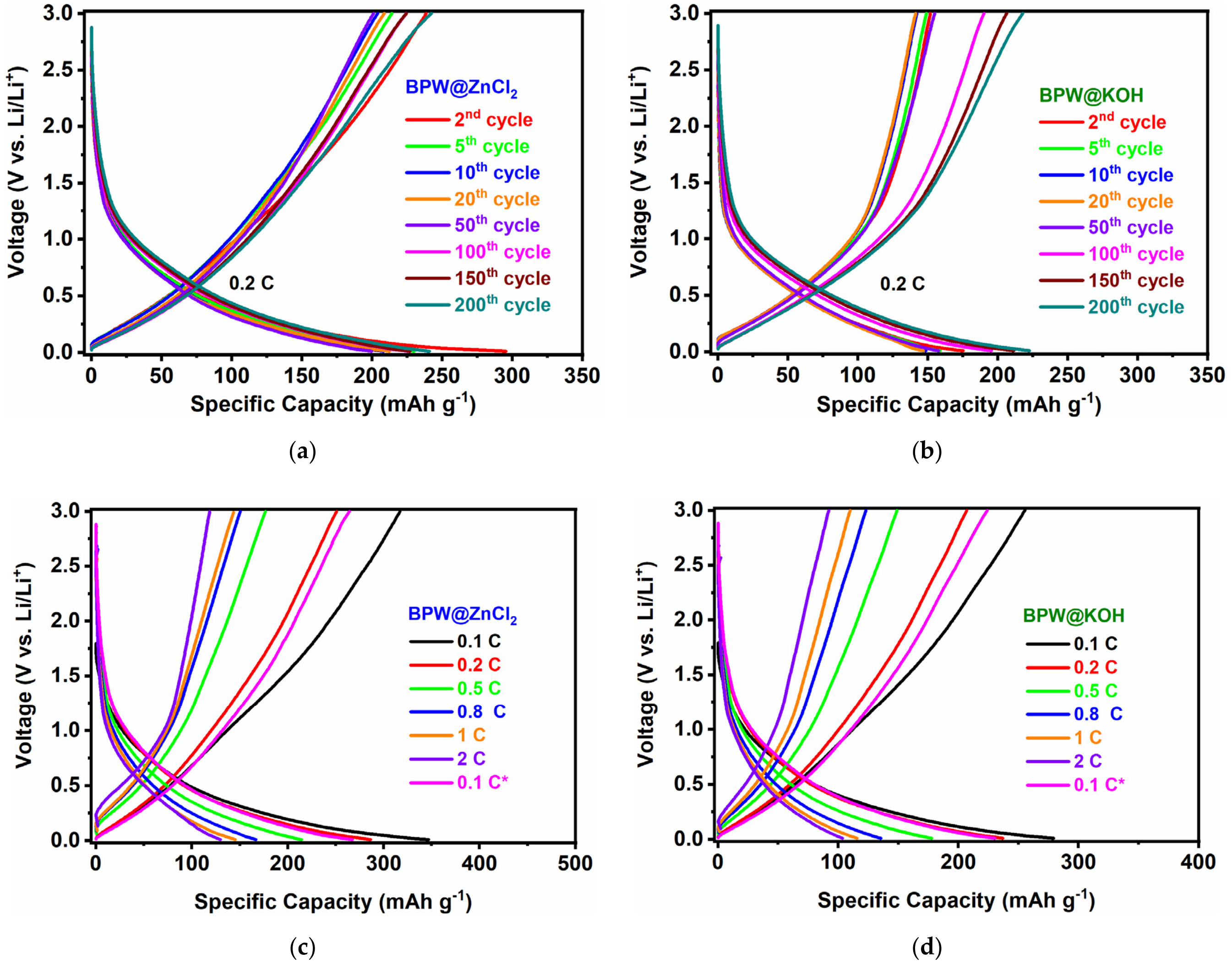

Figure S6. Galvanostatic charge-discharge curves from 2nd to 250th cycle at 0.2 C for (**a**) BPW@ZnCl$_2$, (**b**) BPW@KOH. Rate capability charge-discharge curves for (**c**) BPW@ZnCl$_2$, (**d**) BPW@KOH.
